# Cotton miR319b-Targeted TCP4-Like Enhances Plant Defense Against *Verticillium dahliae* by Activating *GhICS1* Transcription Expression

**DOI:** 10.3389/fpls.2022.870882

**Published:** 2022-05-20

**Authors:** Pei Jia, Ye Tang, Guang Hu, Yonggang Quan, Aimin Chen, Naiqin Zhong, Qingzhong Peng, Jiahe Wu

**Affiliations:** ^1^Hunan Provincial Key Laboratory of Plant Resources Conservation and Utilization, College of Biology and Environmental Sciences, Jishou University, Jishou, China; ^2^State Key Laboratory of Plant Genomics, Institute of Microbiology, Chinese Academy of Sciences, Beijing, China; ^3^The Key Laboratory for the Creation of Cotton Varieties in the Northwest, Ministry of Agriculture, Join Hope Seeds Co. Ltd., Changji, China

**Keywords:** *Gossypium hirsutum*, *Verticillium dahlia*, GhTCP4-like, GhICS1, ghr-miR319b

## Abstract

Teosinte branched1/Cincinnata/proliferating cell factor (TCP) transcription factors play important roles in plant growth and defense. However, the molecular mechanisms of TCPs participating in plant defense remain unclear. Here, we characterized a cotton TCP4-like fine-tuned by miR319b, which could interact with NON-EXPRESSER OF PATHOGEN-RELATED GENES 1 (NPR1) to directly activate *isochorismate synthase 1* (*ICS1*) expression, facilitating plant resistance against *Verticillium dahliae*. mRNA degradome data and GUS-fused assay showed that *GhTCP4-like* mRNA was directedly cleaved by ghr-miR319b. Knockdown of ghr-miR319b increased plant resistance to *V. dahliae*, whereas silencing *GhTCP4-like* increased plant susceptibility by the virus-induced gene silencing (VIGS) method, suggesting that *GhTCP4-like* is a positive regulator of plant defense. According to the electrophoretic mobility shift assay and GUS reporter analysis, GhTCP4-like could transcriptionally activate *GhICS1* expression, resulting in increased salicylic acid (SA) accumulation. Yeast two-hybrid and luciferase complementation image analyses demonstrated that *GhTCP4-like* interacts with *GhNPR1*, which can promote *GhTCP4-like* transcriptional activation in *GhICS1* expression according to the GUS reporter assay. Together, these results revealed that GhTCP4-like interacts with GhNPR1 to promote *GhICS1* expression through fine-tuning of ghr-miR319b, leading to SA accumulation, which is percepted by NPR1 to increase plant defense against *V. dahliae*. Therefore, GhTCP4-like participates in a positive feedback regulation loop of SA biosynthesis via NPR1, increasing plant defenses against fungal infection.

## Introduction

Teosinte branched1/Cincinnata/proliferating cell factor (TCP) proteins are plant-specific transcription factor families, whose names are derived from the first characterized members of the plant developmental pathway, TEOSINTE BRANCHED1 (TB1) in *Zea mays*, CYCLOIDEA (CYC) in *Antirrhinum majus*, and PROLIFERATING CELL FACTOR (PCF) in *Oryza sativa* (Cubas et al., 1999; Kosugi and Ohashi, 2002; Nag et al., 2009). TCP transcription factors (TFs) contain a TCP domain with a nonstandard basic helix-loop-helix (bHLH) motif of 59 amino acids, which is responsible for DNA binding and protein-protein interactions (Heim et al., 2003; Nicolas and Cubas, 2016). TCP proteins are classified into two groups: class I and class II. Class I includes the PCF subclade, and class II is further divided into the CIN and CYC/TB1 subclades (Martin-Trillo and Cubas, 2010). Class I and class II proteins preferentially bind to the consensus elements KHGGGVC and GTGGNCCC, respectively (Aggarwal et al., 2010; Daviere et al., 2014). In Arabidopsis, 5 out of 11 class II TCP genes belonging to CIN types, *TCP2, TCP3, TCP4, TCP10*, and *TCP24*, can be targeted by *miR319* (Nath et al., 2003; Palatnik et al., 2003). TCP family members have been reported to play vital roles in many plant growth and development processes, including hormone signal transduction, gametophyte development, seed germination, leaf shape, internode length, and flower development (Aguilar-Martinez et al., 2007; Giraud et al., 2010; Liu et al., 2018).

Recent studies have shown that TCP proteins participate in plant immunity (Kim et al., 2014; Bao et al., 2019). For example, effectors from multiple pathogens can target TCP TFs to affect plant immune responses (Mukhtar et al., 2011; Wessling et al., 2014). TCP TFs interact with SUPPRESSOR OF rps4-RLD1 (SRFR1, a negative immune regulator), contributing to effector-triggered immunity (Kim et al., 2014). Arabidopsis TCP8, TCP9, and other TCP proteins co-ordinately regulate *ISOCHORISMATE SYNTHASE 1* (*ICS1*) expression to determine salicylic acid (SA) biosynthesis (Wang et al., 2015). The type III effector HopBB1 targets Arabidopsis TCP14 to promote its degradation, leading to increased disease susceptibility (Yang et al., 2017). Arabidopsis TCP15 interacts with MODIFIER OF snc1-1 (MOS1) to participate in plant immune response during cell cycle progression (Zhang et al., 2018). However, the key TCP TFs, especially Class II proteins, that function in plant immunity remain to be identified.

SA plays a key role in plant immunity and induces systemic acquired resistance (SAR) (Fu and Dong, 2013). In plants, two SA biosynthesis pathways use chorismate as the initial substrate via a series of enzymatic reactions with phenylalanine ammonia-lyase (León et al., 1995; Mauch-Mani and Slusarenko, 1996; Coquoz et al., 1998; Ribnicky et al., 1998) and ICS (Verberne et al., 2000; Wildermuth et al., 2001). The ICS-dependent SA biosynthesis pathway is the major pathway (Catinot et al., 2008; Lee et al., 2010; An and Mou, 2011). *ICS1* can be activated in plants infected with pathogens to promote SA accumulation, which acts a major defense regulator non-expressor of PR gene 1 (NPR1), leading to the growth-to-defense transition (Zhang and Li, 2019). Accumulating evidence suggests that *ICS1* expression may be an early regulatory node of the network in plant immunity. For instance, SAR-deficient 1 (SARD1), calmodulin-binding protein 60 g (CBP60g), and WRKY28/46 are positive regulators of Arabidopsis *ICS1* expression, whereas ethylene insensitive 3 (EIN3), EIN3-like1 (EIL1), MUR3, and NAC (for NAM, ATAF1, 2, and CUC2) 19/NAC55/NAC72 negatively regulate *ICS1* expression (Verberne et al., 2000; Tedman-Jones et al., 2008; Chen et al., 2009; Zhang et al., 2010b; Van Verk et al., 2011; Zheng et al., 2012). NPR1 is a key regulator of SA signaling and is required for the regulation of *ICS1* expression (Zhang et al., 2010a). Recently, Arabidopsis TCP8 was confirmed to be a direct positive regulator of *ICS1* expression during an immune response (Wang et al., 2015). Therefore, a sophisticated network regulates *ICS1* expression. Nevertheless, novel regulators of *ICS1* expression remain to be explored in plant immunity.

In Arabidopsis, NPR1 is required for SA to induce *PR1, PR2*, and *PR5* expression (Cao et al., 1994, 1997). NPR1 lacks a DNA-binding domain to interact with DNA, but it contains two protein-protein interaction domains that act as cofactors that interact with TFs to participate in SA-related downstream gene expression (Rochon et al., 2006; Boyle et al., 2009). For example, the interaction between NPR1 and TGAs regulates *PR1* expression (Fan and Dong, 2002). Recently, NPR1 was shown to interact with three class I TCPs. TCP8, TCP14, and TCP15 enhance downstream gene expression, redundantly contributing to SAR establishment (Li et al., 2018). However, NPR1 interaction with other TCPs, especially class II TCPs, remains elusive.

Cotton is an important cash crop that provides natural fiber, plant oil, and protein feedstuff (Zhang et al., 2015). However, the Verticillium wilt caused by *Verticillium dahliae* resulted in huge losses to cotton production every year in China (Klimes et al., 2015). Identification and application of defense genes are important in cotton breeding. Here, we characterized cotton TCP4-like, fine-tuned by ghr-miR319b, that interacts with NPR1 to co-ordinately activate *GhICS1* expression to promote SA accumulation, a feedback loop of SA biosynthesis that is percepted by NPR1 to enhance plant resistance to *V. dahliae* infection. When plants are infected by this fungus, GhTCP4-like can act as a key protein responsible for SA biosynthesis, possibly regulating the transition from plant growth to defense.

## Materials and Methods

### Plant Materials and Growth Condition

The *V. dahliae-*resistant *G. hirsutum* cv. Zhongzhimian 2, susceptible variety Jimian 11, and general resistant variety CCRI 35 were used to analyse the expression of *GhTCP4-like* and ghr-miR319b after *V. dahliae* infection. CCRI 35 was used in VIGS, STTM, and other experiments. Cotton plants were cultivated in a greenhouse with a photoperiod of 16-h-light/8-h-dark at 28/25°C (light/dark) and ~70% humidity.

*Nicotiana benthamiana* plants were grown in pots with 80% nutrient soil and 20% vermiculit with a photoperiod of 16-h-light/8-h-dark at 25°C with 70% humidity.

### Gene Cloning, Multiple-Sequence Alignment, and Phylogenetic Analysis

The coding sequence of *GhTCP4-like* and precursor sequence of ghr-miR319b was amplified from cotton cDNA and genomic DNA, respectively. The MEGA7 program was used for evolutionary tree construction using the neighbor-joining method.

### Gene Expression Analysis

To determine the expression levels of *GhTCP4-like* and ghr-miR319b in different cotton tissues, the roots, stems, and leaves of cotton were harvested at the three-leaf stage and stored at −80°C after quick freezing with liquid nitrogen. The cotton plants were inoculated with *V. dahliae* strain V991 by the root-dipped method at the three-leaf stage **(**Gao et al., 2010), and the control group was treated with sterile water. Roots from six individual seedlings of each treatment were collected at 0, 1, 4, 7, 10, and 13 dpi, and gene expression levels were measured after all samples were collected.

### RNA Extraction, Reverse Transcription and Real-Time Quantitative PCR (qPCR)

Total RNA was extracted using the RNeasy Plant Mini Kit (TSINGKE, Beijing, China), and miRNA was extracted using miRcute (TIANGEN, Beijing, China). RNA concentration was determined using a NanoDrop spectrophotometer (Thermo Fisher Scientific). Two micrograms of total RNA were used for reverse transcription using First-Strand cDNA Synthesis SuperMix (TransGen Biotech, Beijing, China). The products were diluted 10-times, 2 μl of which was used for qPCR experiments with Green qPCR SuperMix UDG (TransGen Biotech, Beijing, China) using the Light Cycler 480 system (Bio-Rad, Foster City, USA). As previously described, stem-loop qPCR was performed for miRNA quantification (Varkonyi-Gasic et al., 2007). All experiments were repeated three times independently, with biological and technical repeats. *GhUB7* was used as an internal control for coding genes, and *GhU6* was used as an internal control for miRNA quantification. Relative gene expression levels of three biologically independent samples were evaluated using the 2^−ΔΔCT^ method (Livak and Schmittgen, 2001). Primers used in this study are listed in Supplementary Table 1.

### 5′ Rapid Amplification of CDNA Ends Assay (5′-RACE)

To confirm that ghr-miR319b directly cleaved the *GhTCP4-like* transcript at 980-nt, we performed a 5′-RACE assay. In brief, leaves of two-week-old cotton plants inoculated with *V. dahliae* for 4 d were harvested. Total RNA was extracted, and the 5′-RACE assay was performed with a HiScript-TS 5′/3′ RACE Kit (Vazyme, Nanjing, China) according to the manufacturer's instructions.

### GUS Staining and Activity Assay

For the effector construct, the coding sequences of *GhTCP4-like* and the target mutant forms of *GhTCP4-like (GhTCP4-like*^*m*^*)* were cloned into plasmid pBI121 and fused with *GUS* under the control of the CaMV 35S promoter. *GhMIR319b* was amplified from cotton genomic DNA and inserted into the vector pCAMBIA1300 to generate the effector construct. All plasmids were electro-transformed into the *Agrobacterium tumefaciens* strain GV3101. Equal amounts of *A. tumefaciens* cultures carrying different effector or reporter vectors were mixed (OD_600_ = 0.8) and injected into the *N. benthamiana* leaf cells. GUS staining was performed as previously described using X-GLUC substrate (Sigma) (Jefferson et al., 1987). GUS enzyme activity in the different treatments was determined using the 4-methylumbelliferyl-β-D-glucuronide (4-MUG) substrate.

### Virus-Induced Gene Silencing (VIGS) and Short Target Tandem Mimic (STTM)

Cotton leaf crumple virus (CLCrV) and tobacco rattle virus (TRV)-induced silencing systems have been employed to knock down *GhTCP4-like* **(**Liu et al., 2002; Gu et al., 2014**)**. A 300 bp fragment specific to *GhTCP4-like* was cloned into pCLCrVA and pTRV2 to generate pCLCrV*-*GhTCP4-like and pTRV*-*GhTCP4-like constructs, respectively. For the STTM assay, two imperfect target sequences for ghr-miR319b were separated using a 48-nt artificial designed linker and cloned into plasmids CLCrVA and pTRV2e, respectively. All plasmids were transformed into *A. tumefaciens strain* GV3101, One-week-old cotton seedlings were used for infection experiments, according to a previous report **(**Gao et al., 2011**)**. At least 30 seedlings were injected with each construct. Silence efficiency was detected ~2 weeks after the injection. Successfully silenced plants were used in subsequent disease resistance analyses. Primers used in this study are listed in Supplementary Table 1.

### Pathogen Activation, Infection, and Disease Assay

The *V. dahliae* strain V991, a highly aggressive defoliating pathogen, was used, and activation and infection assays were performed as previously described (Hu et al., 2018). In brief, V991 was cultured on PDA for 4–5 d at 25°C; the mycelium was picked into Czapek's medium for 5–7 d, after gauze filtration, the spore concentration was adjusted to 10^6^ spores per ml with ddH_2_O. Cotton plants were inoculated using the root-dipped method as previously described (Gao et al., 2010). Disease rate and index were calculated according to a previous report (Cheng et al., 2016). Fungal recovery culture experiments were performed as previously described (Tang et al., 2019). The relative fungal biomass was determined by qPCR assay as previously reported (Xiong et al., 2021).

### Total SA Extraction and Measurement

The roots of *TRV:00* and *GhTCP4-like* plants 1 d after *V. dahliae* inoculation were used to measure total SA content by high-performance liquid chromatography-tandem mass spectrometry (HPLC/MS/MS) system (AB SCIEX QTRAP 4500, Foster, CA, USA) according to a previously described protocol (Xiong et al., 2021). In brief, approximately 200 mg of root samples from corresponding plants were ground to powder with liquid nitrogen and extracted twice with pre-cold 90% methanol overnight at 4°C, centrifuged at 13,000 rpm for 15 min, and the supernatant was dried with nitrogen using a speedvac and then dissolved in 300 μL of 100% methanol. After filtering through a 0.22 μm size filter membrane, total SA was detected by HPLC/MS/MS, and pure SA (Sigma, St. Louis, MO, USA) was used as an external reference to establish the standard curve.

### Yeast Two-Hybrid (Y2H) Assay

For Y2H assays, the coding sequences of *GhTCP4-like* and *GhNPR1* were cloned into plasmids pGADT7 and pGBKT7, respectively, and co-transformed into the yeast strain AH109 using the polyethene glycol (PEG)/LiAc-based method with a yeast transformation kit (TaKaRa, Dalian, China). The monoclonal on SD-/-Trp/-Leu medium was validated by PCR. Positive clones were transferred to SD/-Leu/-Trp/-His/-Ade (with 20 mg/ml X-α-gal) medium to verify the interaction between GhTCP4-like and GhNPR1. The pGBKT7-p53 and pGADT7-largeT pairs were used as positive controls, and pGBKT7-laminC and pGADT7-largeT pairs were used as negative controls (Zhou et al., 2018).

### Luciferase Complementation Imaging (LCI) Assay

The LCI assay was performed according to a previously descripted article (Chen et al., 2008), using an *Agrobacterium*-mediated transient expression system. In brief, the coding sequences of *GhTCP4-like* and *GhNPR1* were cloned into the vectors pCAMBIA1300-CLuc and pCAMBIA1300-NLuc, respectively. The recombinant vectors were then transformed into *A. tumefaciens* strain GV3101. *A. tumefaciens* solution carrying the recombinant plasmid was mixed at a ratio of 1:1. After resting for 3 h at room temperature, the mixture was injected into *N. benthamiana* lower epidermal cells using a sterile, needle-free syringe. After 12 h in the dark, *N. benthamiana* was transferred to a light incubator for 36 h. Luciferase was detected by D-luciferin sodium salt (Solarbio, Beijing, China) using a low-light cooled charge-coupled device camera (Night owl LB985, Germany). *A. tumefaciens* carrying an empty vector mixed with another driven vector was used as a negative control.

### Subcellular Localization

The CDS sequence of *GhTCP4-like* was cloned into the pCAMBIA1302 vector between *Nco* I and *Spe* I upstream of the GFP tag to generate the recombinant plasmid GhTCP4-like-GFP, and the CDS of *AtWRKY22* was fused with mCherry under the control of the CaMV 35S promoter to obtain a nuclear localization marker **(**Wang et al., 2019**)**, which was electrically transferred to *A. tumefaciens* GV3101. After overnight culture, the precipitate was collected by centrifugation and resuspended in MMA (10 mM MES [pH 5.6], 10 mM MgCl_2_, 100 μM acetosyringone) buffer, diluted to an OD_600_ of 0.8, and the either *A. tumefaciens* carrying GFP empty vector or GhTCP4-like-GFP recombinant vector were mixed with *A. tumefaciens* carrying mcherry-tagged fusion vector in equal ratio. After 3 h at room temperature, the bacterial solution was injected into the epidermis of *N. benthamiana* leaves with a sterile syringe; after 12 h in the dark, *N. benthamiana* were transferred to a light incubator for 36 h. Fluorescence signals in the leaf epidermal cells were visualized using a confocal microscope (SP8, Leica, Germany).

### *Cis*-Element Analysis of *GhICS1* Gene Promoter

The sequence 2 kb upstream of the start codon of *GhICS1* was used for *cis*-element analysis by online database Plant CARE [PlantCARE, a database of plant promoters and their cis-acting regulatory elements (ugent.be)].

### Electrophoretic Mobility Shift Assay (EMSA)

The EMSA was performed as previously described **(**Jiao et al., 2020). Briefly, biotin-labeled probes were synthesized by the TSINGKE Biotechnology Company. The full-length coding sequence of *GhTCP4-like* was cloned into the pGEX6p-1vector to generate a GST-tagged recombinant vector and transformed into *E. coli* BL21. After inducible expression with IPTG (0.5 mM) at 28°C, the fusion protein was purified using a GST-tag Protein Purification Kit (Beyotime, Nanjing, China). The Chemiluminescent EMSA Kit (Beyotime, Nanjing, China) was used to visualize the free probe and protein-probe complex, following the manufacturer's instructions.

## Results

### ghr-miR319b and *GhTCP4-Like* Expression Responses to *V. dahliae* Infection

**Figure 1:**
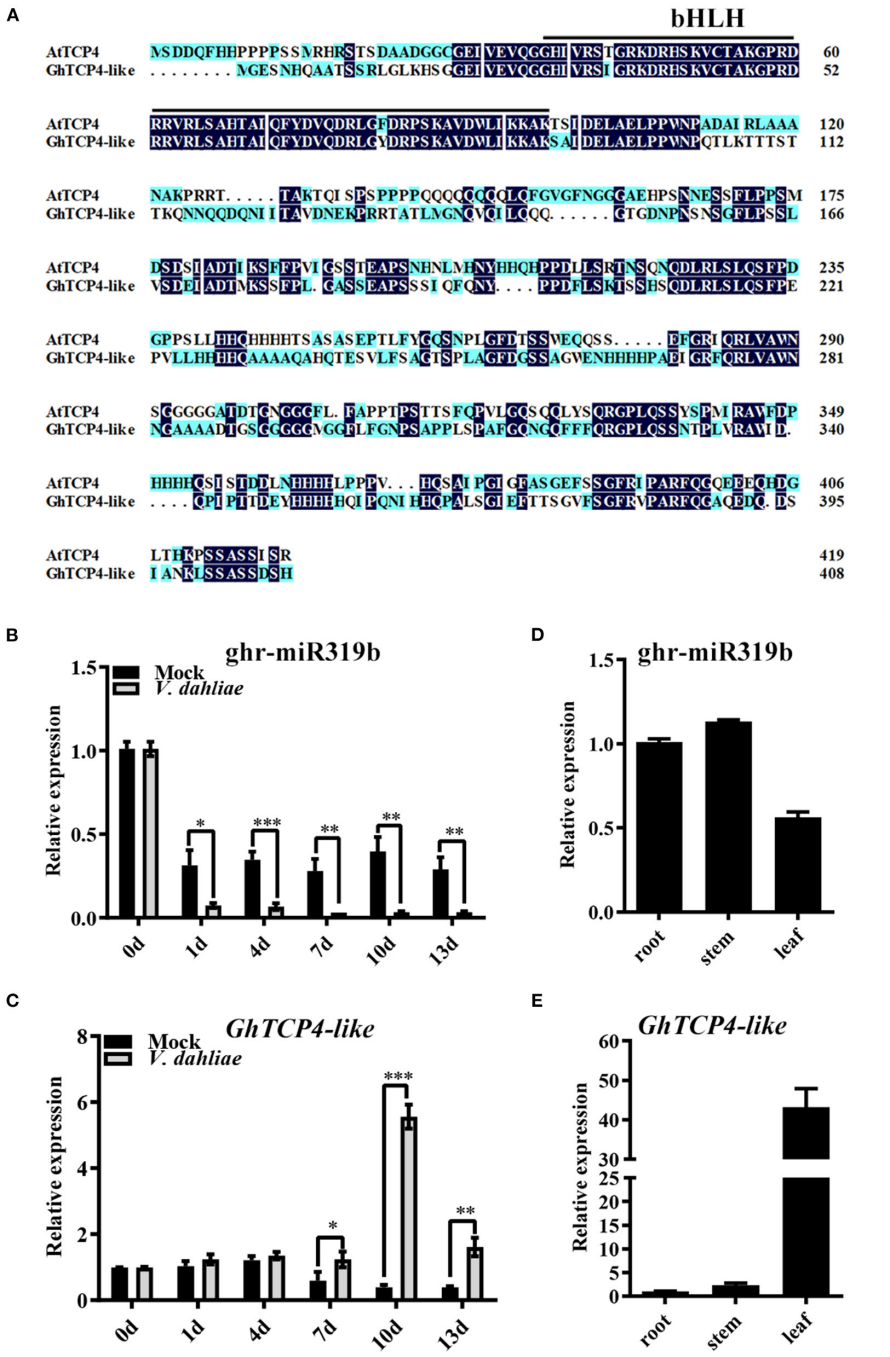
GhTCP4-like characterization and expression patterns of ghr-miR319b and *GhTCP4-like*. **(A)** Amino acid sequence alignment of GhTCP4-like and AtTCP4. The solid black underline indicates the TCP domain (a non-standard bHLH domain). **(B,C)** qPCR analysis of ghr-miR319b and GhTCP4-like expression levels at 0, 1, 4, 7, 10, and 13 d after *V. dahliae* infection. Three-leaf stage cotton seedlings cultured in Gamborg's B5 (B5) liquid medium were infected with *V. dahliae* (10^6^ spores/ml) using the root-dipped method. Error bars represent the standard deviation (SD) of three biological replicates. Student's *t*-test was performed, *p < 0.05, **p < 0.01, ***p < 0.001. **(D,E)** qPCR analysis of ghr-miR319b and GhTCP4-like expression levels in different tissues. Error bars represent the SD of three biological replicates.

Our previous research showed that a cotton-known miRNA, ghr-miR319b, showed a significant differential change in plants infected with *V. dahliae* (Hu et al., 2020). ghr-miR319b includes 21 nucleotides (5′-UUGGACUGAAGGGAGCUCCCU-3′), whose precursor is similar to Arabidopsis *MIR319b* (Supplementary Figure 1A). ghr-miR319b is located at the 3′ terminus of *GhMIR319b* and possesses a typical stem-loop structure (Supplementary Figure 1B). Previous mRNA degradome data have documented that the target gene of ghr-miR319b is Gh_A13G1272 (Supplementary Figure 1C) (Hu et al., 2020). According to amino acid sequence alignment and phylogenetic tree analysis, the Gh_A13G1272 protein showed high identity with Arabidopsis TCP4 (Supplementary Figure 1D). In the *Gossypium hirsutum* genome, the Gh_A13G1272 protein has high homology with Gh_D13G1576 with 99.02% identity (Supplementary Figure 2). Therefore, they can be regarded as potential genes for research. GhTCP4-like contains a nonstandard bHLH domain belonging to the CIN subgroup of Class II TCP family proteins (Figure 1A).

Given the prolonged response of ghr-miR319b to *V. dahliae* infection reported by Hu et al. (2020), we performed qPCR analysis to confirm the response from the beginning of inoculation to 13 days post-inoculation (dpi). In *G. hirsutum* elite cultivar “CCRI 35,” the expression profile of ghr-miR319b was significantly lower when infected by *V. dahliae* than the mock treatment over time (Figure 1B). Notably, the target gene *GhTCP4-like* showed higher expression levels in plants infected with this fungus after 7 dpi compared to mock treatment, seemingly exhibiting a contrary trend with ghr-miR319b accumulation (Figure 1C). The same experiment was performed for the other two cotton cultivars, “Zhongzhimian (ZZM) 2” with high resistance and “Jimian (JM) 11,” which are highly susceptible to *V. dahliae*. As shown in Supplementary Figure 3, ghr-miR319b accumulation and *GhTCP4-like* expression levels in “ZZM 2” and “JM 11” were similar, consistent with “CCRI35” response to *V. dahliae* infection. ghr-miR319b and *GhTCP4-like* genes were constitutively expressed in roots, stems, and leaves (Figures 1D,E). These results suggest that ghr-miR319b regulates *GhTCP4-like* expression, which participates in the plant response to *V. dahliae* infection.

### *GhTCP4-Like* mRNA Was Directedly Cleaved by ghr-miR319b

**Figure 2:**
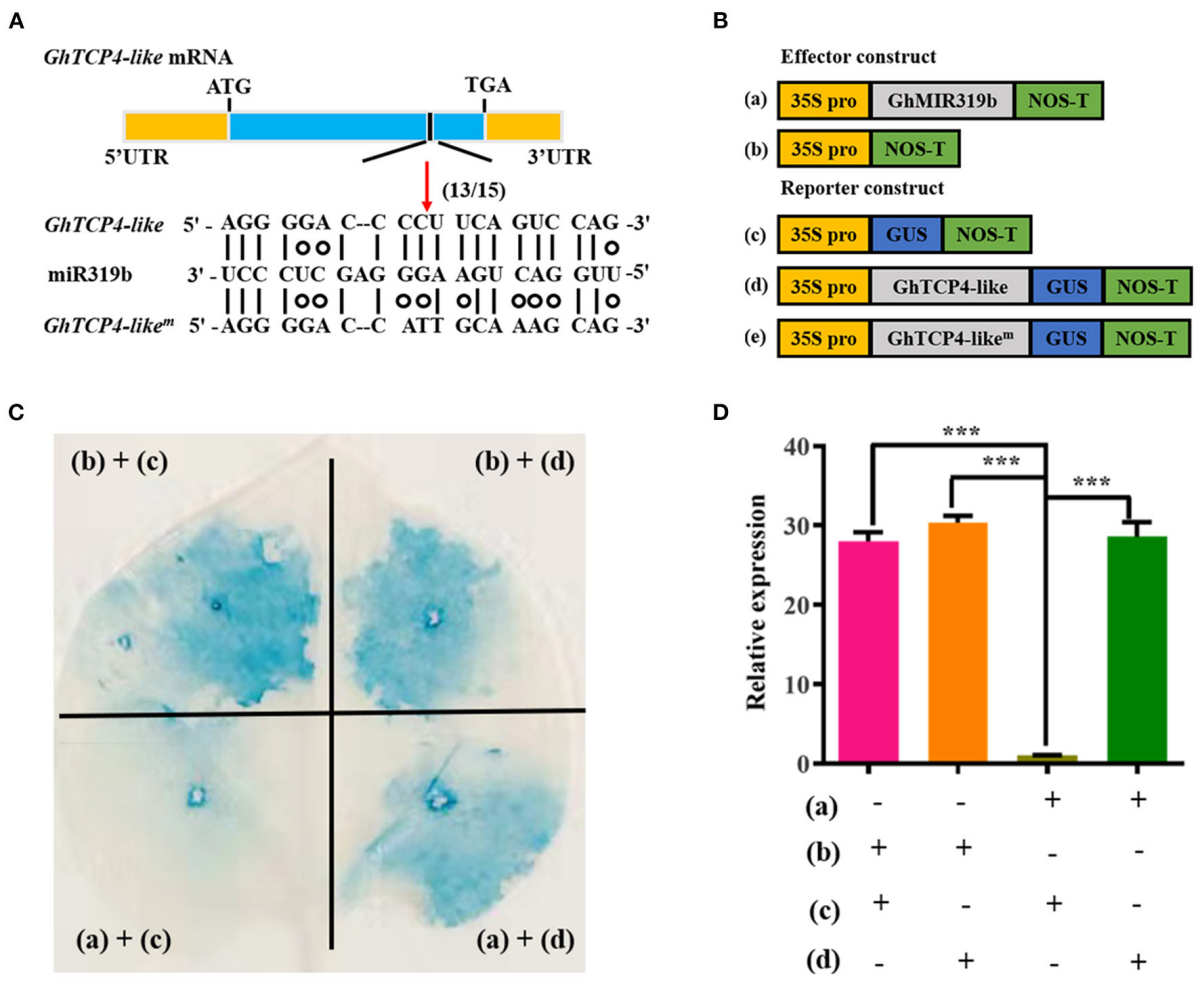
*GhTCP4-like* directedly cleaved by ghr319b. **(A)** Diagram of the structure of *GhTCP4-like* transcript and 5′-RACE analysis. *GhTCP*4−*like*^m^ is an equivocal mutation sequence of *GhTCP4-like* mRNA on the ghr-miR319b target sequence. The black box points to the predicted cleavage site at 980-nt of *GhTCP4-like* mRNA. (13/15) represents that 13 out of 15 clones are cleaved in target sequence by sequencing. **(B)** Schematic diagram of the constructs for transient expression assay. (a), (b), (c) and (d) represent 35S:GhMIR319b, pCAMBIA1300, 35S:GhTCP4-GUS and 35S:GhTCP4-like^m^-GUS, respectively. **(C)** GUS staining assay. The combinations of *A. tumefaciens* containing vectors labeled in the edge indicated in **(B)** were co-injected into the tobacco leaf cells. **(D)** *GhTCP4-like* relative expression in treated leaf spots as indicated in **(C)**. Error bar means SD of three independent biological replications. Student's *t*-test was performed, ***p < 0.001. Leaves were harvested 48 h after agroinfiltration. All experiments were replicated at least three times.

Our previous study showed that *GhTCP4-like* mRNA was first recognized by ghr-miR319b through base complementarity and was directedly cut at 980-nucleotide (nt) according to mRNA degradome sequencing data (Supplementary Figure 1C). A 5′-RACE analysis was performed to confirm *GhTCP4-like* transcript cleavage using the ghr-miR319b guide. Sequencing analysis revealed that 13 out of 15 clones of *GhTCP4-like* mRNA were cleaved at the 980-nt through sequencing analysis (Figure 2A). GUS-fused reporter analysis was conducted to verify the cleavage of the *GhTCP4-like* transcript directed by ghr-miR319b. The *GhTCP4-like* coding sequence (CDS) and the corresponding mutant in the target sequence were fused with the *GUS* gene under the control of the CaMV 35S promoter and were constructed as reporter vectors, 35S:GhTCP4-like-GUS and 35S:GhTCP4-like^m^-GUS. *GhMIR319b* was inserted into the plant expression vector under control of the CaMV 35S promoter as an effector vector, 35S:GhMIR319b (Figure 2B). Co-expression analysis in *Nicotiana benthamiana* leaf cells was performed using the agroinfiltration technique. As shown in Figure 2C, the leaves agroinfiltrated with 35S:GhTCP4-like-GUS or 35S:GhTCP4-like^m^-GUS showed normal blue staining by GUS. The tobacco leaves equally infiltrated with 35S:GhMIR319b and 35S:GhTCP4-like-GUS showed a weak blue color, whereas the leaves treated with 35S:GhMIR319b and 35S:GhTCP4-like^m^-GUS exhibited normal blue color. The GUS staining analysis revealed that the expression levels of *GhTCP4-like* in various leaf spots treated with combinations of vectors (Figure 2C) showed that *GhTCP4-like* can be directedly cleaved by ghr-miR319b in tobacco cells (Figure 2D). These results demonstrated that *GhTCP4-like* mRNA can be directedly cleaved by ghr-miR319b through a post-transcriptional process.

### ghr-miR319b-*GhTCP4*-*Like* Module Regulates Plant Resistance to *V. dahliae*

**Figure 3:**
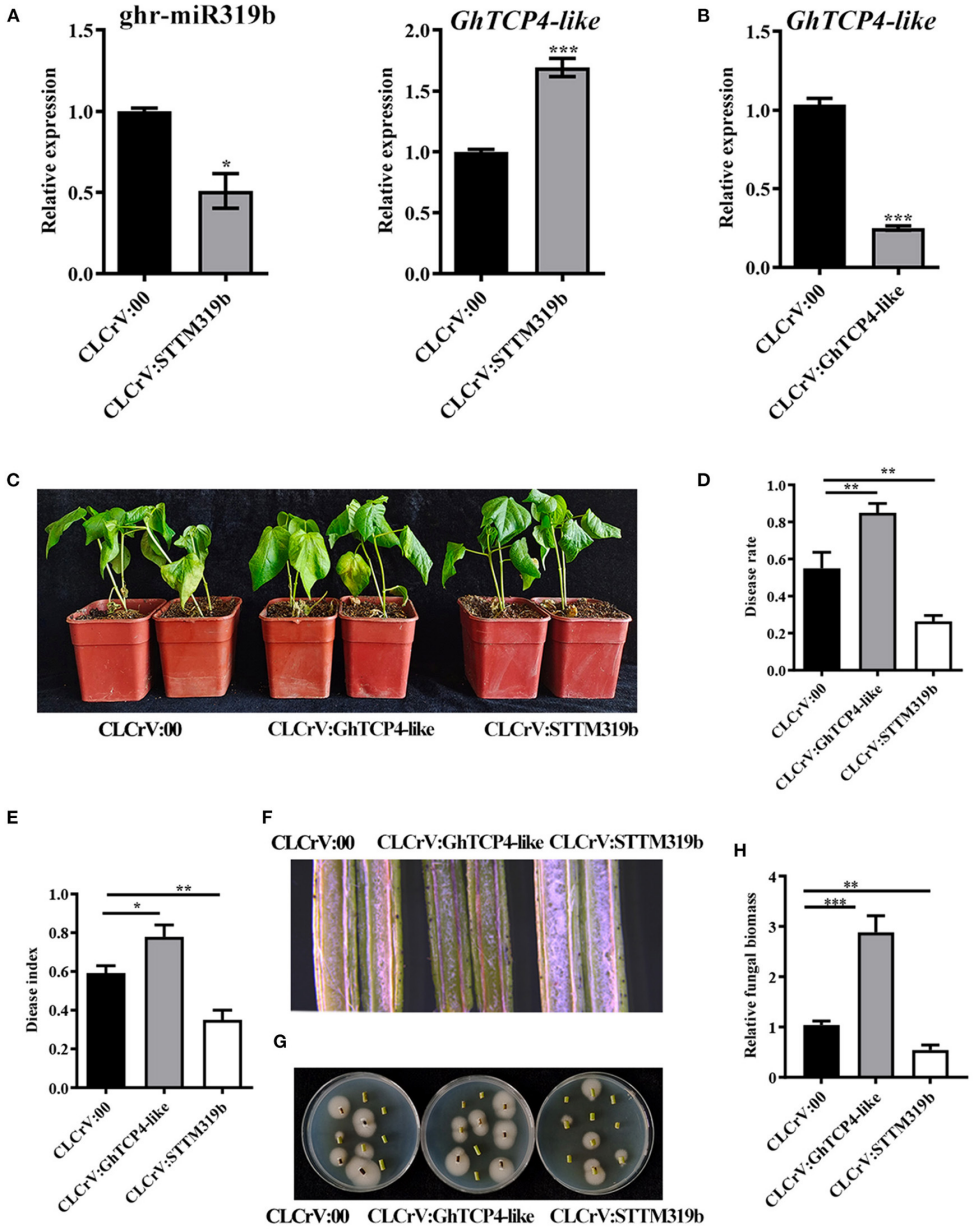
Resistance analysis of ghr-miR319b and *GhTCP4-like* against *V. dahlia*. **(A)** Transcript level of ghr-miR319b and *GhTCP4-like* in the control and STTM plants determined by qPCR. Total RNA was extracted from the second true leaf 14 d after agroinfiltration. Error bar means SD of three independent biological replications. Student's *t*-test was performed, *p < 0.05, ***p < 0.001. **(B)** The transcript level of *GhTCP4-like* in control and *GhTCP4-like*-silenced plants was determined by qPCR. Error bar means SD of three independent biological replications. Student's *t*-test was performed, ***p < 0.001. **(C)** Disease symptom of CLCrV:00, CLCrV:GhTCP4-like and CLCrV:STTM319b plants were photographed at 14 dpi. **(D,E)** Disease rate and disease index were calculated at 14 dpi. More than 30 plants per treatment were sampled. Error bar means SD. Student's *t*-test was performed, *p < 0.05, **p < 0.01. **(F)** Color intensity of longitudinal sections of the stem of CLCrV:00, CLCrV:GhTCP4-like and CLCrV:STTM319b plants 14 dpi. Photos were taken by the body microscope. **(G)** Fungal discovery culture assay. *V. dahliae* growth condition on PDA medium from stems of CLCrV:00, CLCrV:GhTCP4-like, CLCrV:STTM319b plants 14 dpi. Photographs were taken after 5 days of culture at 25°C. **(H)** The relative fungal biomass assay. Stems from CLCrV:00, CLCrV:GhTCP4-like, CLCrV:STTM319b plants were used to extract DNA at 14 dpi, which were used as templates for relative fungal biomass analysis by qPCR. Error bar means SD of three independent biological replications. Student's *t*-test was performed, **p < 0.01, ***p < 0.001.

To elucidate the function of ghr-miR319b and *GhTCP4-like* in the plant response to *V. dahliae* infection, ghr-miR319b- and *GhTCP4-like*-silenced plants were generated using the VIGS method, which is a robust tool for identifying the function of genes associated with plant defense and development, especially at the juvenile stage. Cotton leaf crumple virus (CLCrV)-derived vectors and/or short tandem target mimic (STTM) technology were used to develop ghr-miR319b-silenced plants (CLCrV:STTM319b) and *GhTCP4-like*-silenced plants (CLCrV:GhTCP4-like). *Phytoene desaturase* (*PDS*)-silenced plants showed a photobleaching phenotype as a VIGS marker about 14 d post-agroinfiltration (Supplementary Figure 4) upon examining ghr-miR319b accumulation and the level of *GhTCP4-like* expression. The results showed that ghr-miR319b accumulation in silenced plants was significantly reduced by 50% compared to plants agroinfiltrated with empty vectors (CLCrV:00, hereafter referred to as the control), in which *GhTCP4-like* expression levels were significantly higher (Figure 3A). *GhTCP4-like* expression in *GhTCP4-like*-silenced plants was significantly decreased by 74% compared to control (Figure 3B).

To evaluate ghr-miR319b-*GhTCP4-like* function in plant defense, the VIGS plants were inoculated with *V. dahliae*. As shown in Figure 3C, ghr-miR319b-silenced plants showed higher resistance to this fungus than the control with less yellow and wilt leaves, whereas *GhTCP4-like*-silenced plants exhibited higher susceptibility to *V. dahliae* infection, possessing more yellow and wilt leaves. Statistical analysis showed that the disease rate and disease index in CLCrV:STTM319b plants were lower than those in control, whereas, they were higher in CLCrV: GhTCP4-like plants (Figures 3D,E). Stem longitudinal sections in ghr-miR319b-silenced plants were lighter than those in control, indicating less damage. However, longitudinal sections of the stems in *GhTCP4-like*-silenced plants were heavier than those in the control (Figure 3F). Fewer fungal colonies were recovered from stem sections of ghr-miR319b-silenced plants in the media compared to the control, while more fungal colonies appeared from stem sections of *GhTCP4-like-*silenced plants, which was supported with the results of relative fungal biomass assay (Figures 3G,H). To further verify ghr-miR319b-*GhTCP4-like* the function in plant resistance to *V. dahliae* infection, a parallel experiment was performed using another set of tobacco rattle virus (TRV)-induced gene silencing (TRVIGS) plants. As with the results of CLCrV-derived VIGS (CLCrVIGS) plants, TRVIGS plants with ghr-miR319b knockdown showed higher resistance to this fungus compared to control (TRV:00), while those with *GhTCP4-like* knockdown showed significantly increased susceptibility (Supplementary Figures 5, 6). The above results demonstrate that *GhTCP4-like* fine-tuned by ghr-miR319b is a positive regulator of plant resistance to *V. dahliae* infection.

**Figure 4:**
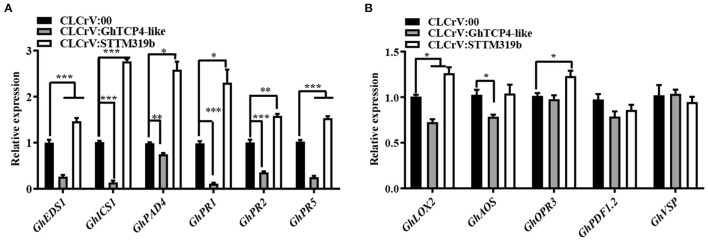
Expression levels of SA- and JA-related genes in CLCrV:00, CLCrV:GhTCP4-like and CLCrV:STTM319b plants under *V. dahliae* infection. **(A,B)** The expression analysis of SA biosynthesis-related genes (*GhEDS1, GhICS1* and *GhPAD4*) and SA responsive genes (*GhPR1, GhPR2*, and *GhPR5*) in various plants 1-day post-inoculation (dpi). **(B)** The expression analysis of JA biosynthesis-related genes (*GhLOX2, GhAOS*, and *GhOPR3*) and JA responsive genes (*GhPDF1.2* and *GhVSP*) in various plants 1 dpi. Root samples were collected from various treatments. Error bar means SD of three independent biological replications. Student's *t*-test was performed, *p < 0.05, **p < 0.01, ***p < 0.001.

To elucidate the molecular mechanism of ghr-miR319b-*GhTCP4-like* module function in plant's response to *V. dahliae*, we tested whether this module affects SA- and JA-related gene expression levels. The results showed that three SA-related synthesis genes, *GhICS1, GhEDS1*, and *GhPAD4*, were significantly upregulated in ghr-miR319b-silenced plants compared to the control under *V. dahliae* infection, whereas they were significantly downregulated in *GhTCP4-like*-silenced plants (Figure 4A). Three other SA response genes, *GhPR1, GhPR2*, and *GhPR5*, showed higher expression levels in ghr-miR319b-silenced plants than in control and exhibited lower expression levels in *GhTCP4-like*-silenced plants (Figure 4A). The results suggested that *GhTCP4-like* knockdown possibly reduced SA biosynthesis by repressing expression of *GhICS1, GhEDS1* and *GhPAD4*, leading to down-regulation of *GhPR1, GhPR2* and *GhPR5* under the pathogen infection. However, the JA-related genes *GhAOS, GhLOX2, GhOPR3, GhPDF1.2*, and *GhVSP* showed slight changes in expression levels among ghr-miR319b-silenced plants, *GhTCP4-like*-silenced plants, and the control inoculated with *V. dahliae* (Figure 4B), suggesting that the ghr-miR319-*GhTCP4-like* module participates in the plant response to this fungal infection and is not involved in the JA signaling pathway. These results suggest that the ghr-miR319-*GhTCP4-like* module is associated with SA synthesis and signaling pathways that regulate plant resistance to *V. dahliae* infection.

### GhTCP4-Like Regulates *GhICS1* Expression for Mediating SA Accumulation

**Figure 5:**
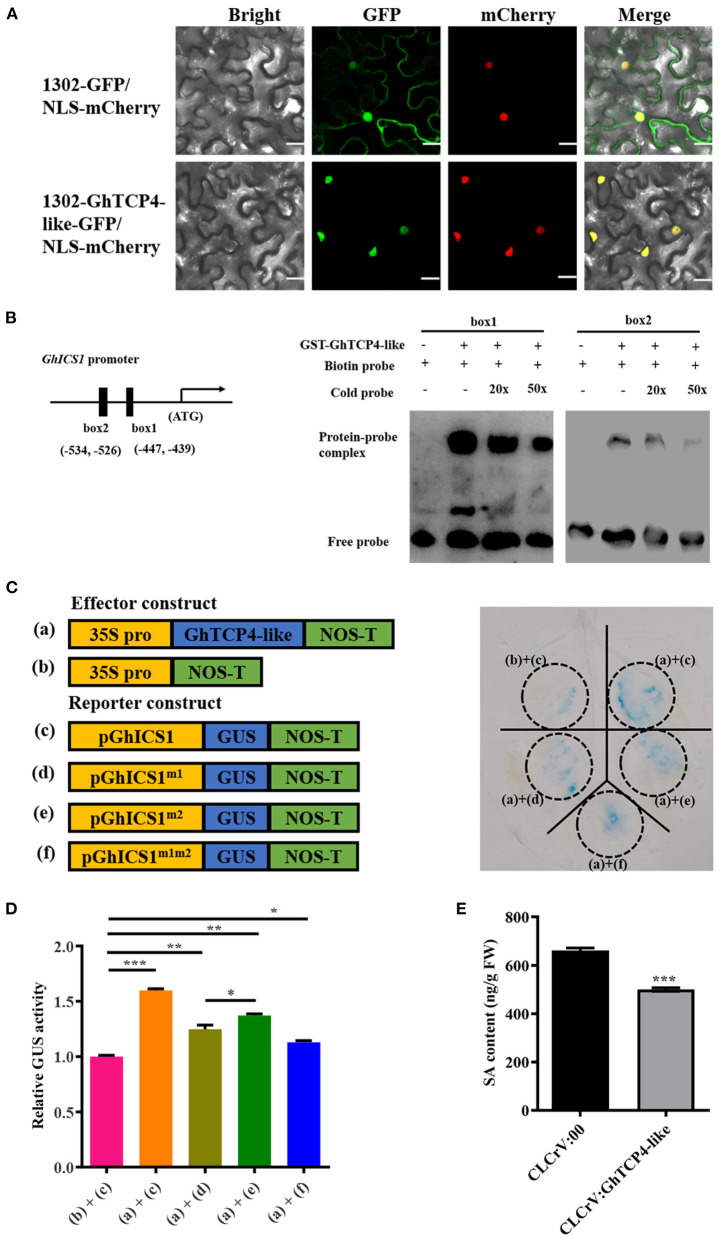
GhTCP4-like directly transactivates *GhICS1* expression. **(A)** Subcellular localization of GhTCP4-like in tobacco leaf cells. NLS-mCherry was a nuclear location marker. Scale bar = 20 μm. **(B)** Electrophoretic mobility shift assay (EMSA). Structure of *GhICS1* promoter in the left panel. Solid black boxes indicate predicted two GhTCP4-like binding sites (GTGGCACC) in the *GhICS1* promoter. GhTCP4-like bound to GTGGCACC motif from *GhICS1* promoter in the right panel. The lower bands represent unbound probes; lagging bands indicate the complexity of protein and probe. **(C)** GUS reporter analysis for GhTCP4-like transactivating *GhICS1* expression. Schematic diagram of the constructs for GUS reporter assays in tobacco leaf cells in the left panel. (a), (b), (c), (d), (e), and (f) represent p35S:GhTCP4-like, pCAMBIA1300, pGhICS1:GUS, pGhICS1^m1^:GUS, pGhICS1^m2^:GUS and pGhICS1^m1m2^:GUS, respectively. Tobacco leaves were harvested at 48 h after agroinfiltration. **(D)** GUS enzyme activity assayed by 4-MU measurement of treated leaf spots indicated in **(C)**. “+” indicates that the corresponding treatment has been added, “–“ indicates that the corresponding treatment has not been added. Tobacco leaves were harvested at 48 h after agroinfiltration. **(E)** Determination of total SA content. SA of *GhTCP4-like*-silenced and control plants 1 d after *V. dahliae* infection was measured by HPLC-MS/MS. Error bar means SD of three independent biological replications. Student's *t*-test was performed, *p < 0.05, **p < 0.01, ***p < 0.001. All experiments were repeated more than three times with similar results.

Given that *GhTCP4-like* knockdown significantly downregulated the expression of SA biosynthesis-related genes, we hypothesized that GhTCP4-like would affect SA biosynthesis. A recent study also reported that Arabidopsis TCP8/9 proteins could co-ordinately regulate *ICS1* expression to participate in plant immunity **(**Wang et al., 2015**)**, which is similar to our results of *GhTCP4-like* knockdown regulating *GhICS1* expression during *V. dahliae* infection (Figure 4A). A subcellular location was investigated using a GFP reporter system in *N. benthamiana* leaf cells to characterize the GhTCP4-like transcription factor. As shown in Figure 5A, the green fluorescence of the GhTCP4-like-GFP protein located in the nucleus of tobacco leaf cells merged with a red color from the nucleus protein mCherry. These results suggested that GhTCP4-like proteins are distributed in the nucleus.

The *cis*-element bond of GhTCP4-like is generally the GTGGNCCC box previously reported (Martin-Trillo and Cubas, 2010), and we found that there are two putative GhTCP4-like binding sequences (GTGGCACC) in the *GhICS1* promoter, namely box1 and box2, located−447 to−439 and−534 to−526 upstream of the translation initiation site (ATG), respectively (Figure 5B). The two fragments containing box1 or box2 and their flanking sequences were chemically synthesized and labeled with biotin at the 5′ terminal end as probes to perform an electrophoretic mobility shift assay (EMSA). The box1/box2 probe incubated with a combination of GhTCP4-like showed one upshifted bond in the gel, which weakened with increasing cold probe levels (Figure 5B). Therefore, box1 and box2 were verified to be specifically bound to GhTCP4-like.

To further elucidate the transcriptional activity of GhTCP4-like to *GhICS1*, a GUS reporter assay in *N. benthamiana* leaf cells was conducted. A 1,200 bp of *GhICS1* promoter was isolated to drive *GUS* gene expression as a reporter vector (pGhICS1:GUS), and the *GhICS1* promoter mutated in box1 and/or box2 was used to control *GUS* gene expression as promoter mutant reporter vectors (pGhICS1^m1^:GUS, pGhICS1^m2^:GUS, and pGhICS1^m1m2^:GUS). *GhTCP4-like* was expressed under the control of the CaMV 35S promoter as an effector vector, p35S:GhTCP4-like (Figure 5C). As shown in Figure 5C, tobacco leaves agroinfiltrated with pGhICS1:GUS showed normal blue staining by GUS, indicating that the *GhICS1* promoter can drive GUS gene expression. When p35S:GhTCP4-like and pGhICS1:GUS were co-infiltrated with tobacco leaves, they were bluer than the leaves agroinfiltrated with pGhICS1:GUS. Tobacco leaves co-injected with p35S:GhTCP4-like and pGhICS1^m1^:GUS or pGhICS1^m2^:GUS were lighter blue than those co-injected with p35S:GhTCP4-like and pGhICS1:GUS. However, there was less blue color in tobacco leaves agroinfiltrated with p35S:GhTCP4-like and pGhICS^m1m2^:GUS, suggesting that box1 and box2 in the *GhICS1* promoter have additive effects on GhTCP4-like activation of *GhICS1* transcription. In line with these results, the GUS activities tested by the 4-MU assay showed that the leaves co-infected with p35S:GhTCP4-like and pGhICS1:GUS had the highest enzyme activity compared to those co-injected with p35S:GhTCP4-like and pGhICS1^m1^:GUS, p35S:GhTCP4-like and pGhICS1^m2^:GUS, and p35S:GhTCP4-like and pGhICS1^m1m2^:GUS (Figure 5D). Notably, the enzyme activity of sample treated with p35S:GhTCP4-like and pGhICS1^m1^:GUS significantly lower than that with p35S:GhTCP4-like and pGhICS1^m2^:GUS, indicated that box1 is more essential than box2 to promote *GhICS1* expression by GhTCP4-like. These results demonstrate that GhTCP4-like can activate *GhICS1* expression through the additive effects of the box1 and box2 *cis*-elements.

Given that GhTCP4-like can directly regulate *GhICS1* expression, we hypothesized that SA levels would be altered when *GhTCP4-like* expression is disrupted. HPLC-MS/MS analysis showed that *GhTCP4-like*-silenced plants had significantly lower SA content than the control (Figure 5E). These data confirm that GhTCP4-like transcriptionally activates *GhICS1* expression, increasing SA content.

### GhNPR1 Interacts With GhTCP4-Like

**Figure 6:**
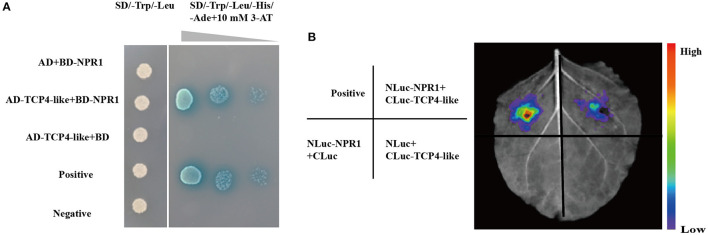
GhTCP4-like interact with GhNPR1. **(A)** Yeast two-hybrid (Y2H) assay shows GhTCP4-like interaction with GhNPR1. All recombination yeast cells were grown on SD/-Trp/-Leu medium. After gradient dilution and sprayed 10 mM 3-AT and 20 mg/ml X-α-Gal on SD/-Trp/-Leu /-His/-Ade medium. Yeast transformed with paired plasmid AD-largeT and BD-p53 were used as a positive control, AD-largeT and BD-laminC were used as a negative control. Photos were taken after 3 d at 28°C. **(B)** LCI assays to detect the association between GhTCP4-like and GhNPR1. Agrobacterium strains containing the indicated vectors were co-expressed in leaf cells of *N. benthamiana*. The luminescence signal was detected by applying 1 mM luciferin after 48 h of agroinfiltration. The positive control was constructed according to Chen et al. (2008). The colored scale indicates the intensity of luciferin activity. All experiments were repeated more than three times with similar results.

To explore whether the regulation of GhTCP4-like to *GhICS1* transcription is associated with other regulators, we need to search for its partners in cells. Several reports have shown that the NPR1 is a partner of TCPs (Li et al., 2018). Therefore, we performed a yeast two-hybrid assay to verify whether GhTCP4-like interactes with GhNPR1. The *GhTCP4-like* coding sequence (CDS) was isolated and inserted into the AD vector, and the *GhNPR1* CDS was constructed into a BD vector. As shown in Figure 6A, yeast co-transformed with GhTCP4-like and GhNPR1 grew on SD/-Trp/-Leu/-His/-Ade medium at different dilution concentrations and was stained blue as well as the positive control, while yeasts treated with BD and AD-TCP4-like, AD and BD-NPR1, or the negative control, did not grow. These results indicated that GhTCP4-like directly interacts with GhNPR1. To confirm the GhTCP4-like interacts with GhNRP1, we conducted a luciferase complementation image (LCI) analysis in tobacco cells. The CDSs of *GhTCP4-like* and *GhNPR1* were fused to the C-terminus and N-terminus of LUC, respectively, generating cLUC-TCP4-like and nLUC-NPR1 vectors. As shown in Figure 6B, leaf spots agroinfiltrated with cLUC-TCP4-like and nLUC-NPR1 showed LUC fluorescence, while other leaf spots injected with various vectors, as indicated in the left panel of Figure 6B did not. The results of the LCI analysis suggested that GhTCP4-like interacts with GhNPR1, similar to the interaction of Arabidopsis TCP8/14/15 with NPR1 reported by Li et al. (2018).

### GhNPR1 Promotes GhTCP4-Like Transcriptional Activation Activity for *GhICS1* Expression

**Figure 7:**
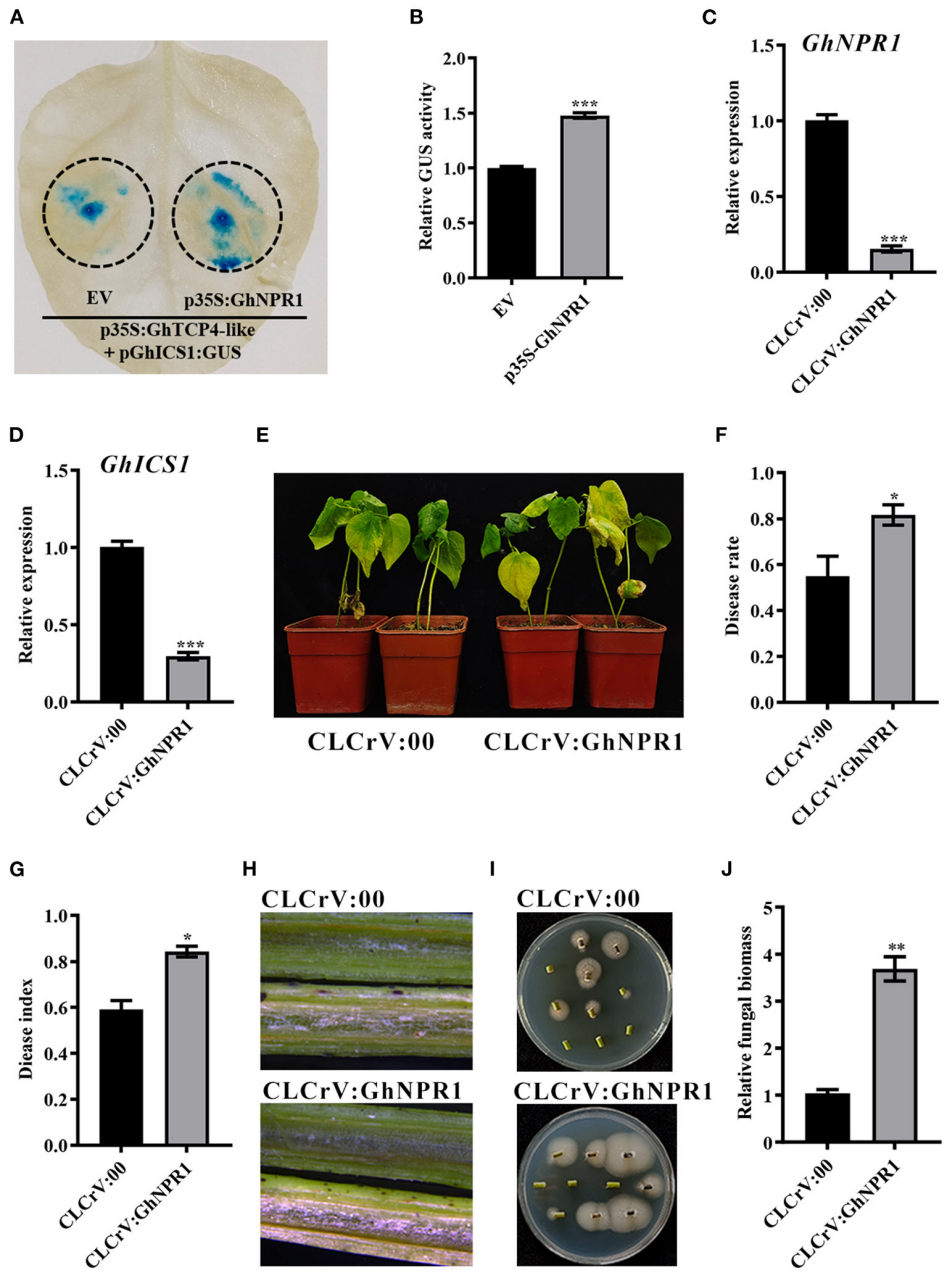
GhNPR1 promotes transcriptional activating activity of GhTCP4-like for *GhICS1* expression. **(A)** GUS staining assay. EV represents pCAMBIA1300. Tobacco leaves were harvested at 48 h after corresponding treatments. **(B)** GUS enzyme activity measurement. Error bar means SD of three independent biological replications. Student's *t*-test was performed, ***p < 0.001. **(C)** *GhNPR1* expression level in CLCrV:00 and CLCrV:GhNPR1 plants. Total RNA was extracted 14 d after agroinfiltration. Error bar means SD of three independent biological replications. Student's *t*-test was performed, *p < 0.05. **(D)** Expression of *GhICS1* in CLCrV:00 and CLCrV:GhNPR1 plants 1 dpi. Error bar means SD of three independent biological replications. Student's *t*-test was performed, ***p < 0.001. **(E)** Disease symptoms of CLCrV:00 and CLCrV:GhNPR1 plants after *V. dahliae* infection. Photos were taken at 21 dpi. **(F,G)** Disease rate and disease index of CLCrV:00 and CLCrV:GhNPR1 plants 21 dpi. Error bar means SD of three independent biological replications. Student's *t*-test was performed, *p < 0.05. **(H)** Color intensity of longitudinal sections of the stem of CLCrV:00 and CLCrV:GhNPR1 plants 21 dpi. Photos were taken by the body microscope. **(I)** Fungus discovery culture assay. Stems from CLCrV:00 and CLCrV:GhNPR1 plants 21 dpi were sprayed on PDA medium. Photos were taken after 5 d of culture at 25°C. **(J)** Relative fungal biomass in CLCrV:00 and CLCrV:GhNPR1 plants were determined by qPCR at 21 dpi. Error bar means SD of three independent biological replications. Student's *t*-test was performed, **p < 0.01.

Because GhNPR1 can interact with GhTCP4-like, we investigated whether GhNPR1 affects GhTCP4-like transcriptional activity in *GhICS1* expression. The *GhNPR1* CDS was cloned into a plant expression vector under the control of the CaMV 35S promoter to carry out GUS reporter analysis. As shown in Figure 7A, the leaf spots co-injected with p35S:GhTCP4-like and pGhICS1:GUS showed a normal blue color under GUS staining treatment, whereas the leaf spots co-injected with p35S:GhNPR1, p35S:GhTCP4-like, and pGhICS1:GUS exhibited a heavier blue color than the former leaf spots. The GUS content tested with 4-MU supported the hypothesis that GhNPR1 promotes GhTCP4-like activation of *GhICS1* transcription (Figure 7B). *GhNPR1*-silenced plants were generated using the VIGS method to elucidate GhNPR1 response to *V. dahliae* infection (Figure 7C). As expected, GhNPR1 knockdown significantly reduced *GhICS1* expression levels compared to control after *V. dahliae* inoculation (Figure 7D). Moreover, *GhNPR1*-silenced plants showed higher susceptibility to *V. dahliae* infection than the control, with more yellow and wilting leaves, higher disease rate, disease index, and fungal biomass in infected stems (Figures 7E–J). Therefore, GhNPR1 co-ordinately participates in plant resistance to pathogen infection via two pathways. First, GhNPR1, one of the receptors, can generally activate SA signaling to increase plant defense against pathogens. Second, GhNPR1 interacts with GhTCP4-like co-ordinately promotes *GhICS1* transcription to increase SA biosynthesis in the feedback regulation of SA biosynthesis.

## Discussion

TCPs are transcription factors that regulate plant growth and development, including gametophyte development, seed germination, leaf shape, internode length, and flower development (Aguilar-Martinez et al., 2007; Giraud et al., 2010; Liu et al., 2018). Recently, accumulating evidence has shown that TCPs play a role in plant immunity against many pathogenic infections (Mukhtar et al., 2011; Wang et al., 2015; Yang et al., 2017; Li et al., 2018). However, it is necessary to identify novel TCPs and reveal their potential mechanisms in plant responses to pathogen infections. In this study, we characterized the cotton TCP4-like functions in plant immunity. *GhTCP4-like* can be directedly cleaved by ghr-miR319b through a post-transcriptional process and activates *GhICS1* transcription and promotes SA biosynthesis, resulting in increased plant resistance to *V. dahliae* infection via GhNPR1 coordination.

GhTCP4-like, a class II protein, was fine-tuned by ghr-miR319 through a post-transcriptional process, which participates in plant response to *V. dahliae* infection. Knockdown of *GhTCP4-like* significantly reduced plant resistance to this fungal infection compared with the control, whereas ghr-miR319b-silenced plants showed a higher resistance. In Arabidopsis, TCP8 and TCP9 participate in plant responses to pathogens (Wang et al., 2015). AtTCP15 regulates plant immune response during cell cycle progression (Zhang et al., 2018). Arabidopsis TCP8, TCP9, and TCP15 belong to class I proteins, while GhTCP4-like is a class II protein and fine-tuned by ghr-miR319b. Therefore, *GhTCP4-like*, targeted by ghr-miR319, is a novel module that participates in plant immunity.

In this study, GhTCP4-like activated *GhICS1* transcription, which promoted SA accumulation and increased the expression levels of SA-related genes, resulting in plant resistance to *V. dahliae* infection. Several transcription factors and regulators have transcriptionally regulated *ICS1* in plant immunity. Four positive regulators, SARD1, CBP60g, and WRKY28/46, have been shown to activate *ICS1* expression in Arabidopsis to promote SA biosynthesis. In contrast, EIN3, EIL1, MUR3, and NAC19/NAC55/NAC72 negatively regulates *ICS1* expression during plant immunity (Verberne et al., 2000; Tedman-Jones et al., 2008; Chen et al., 2009; Zhang et al., 2010b; Van Verk et al., 2011; Zheng et al., 2012). A similar report showed that, in Arabidopsis, TCP8 could directly activate *ICS1* expression during the immune response (Wang et al., 2015). Therefore, our results further explain that *ICS1* transcription is a knot of the network that is regulated by several factors in plant resistance to pathogen infection.

GhTCP4-like interacts with GhNPR1, which co-ordinately regulates *GhICS1* transcription to promote SA biosynthesis. Knockdown of *GhNPR1* significantly reduced *GhICS1* expression, resulting in increased susceptibility to *V. dahliae* infection compared with the control. Similar reports have shown that the NPR1 interacts with TCP proteins to participate in plant immunity. For example, NPR1 interacts with TCP15 to promote its transcriptional activation activity of *PR1* in Arabidopsis but does not participate in SA biosynthesis like GhTCP4-like **(**Li et al., 2018**)**. Therefore, in plants, NPR1 can interact with TCP proteins to regulate *ICS1* transcription to promote SA accumulation or act downstream gene expression, facilitating plant response to pathogen infection partially due to NPR1 and TGA protein interaction to promote defense-related expression.

**Figure 8:**
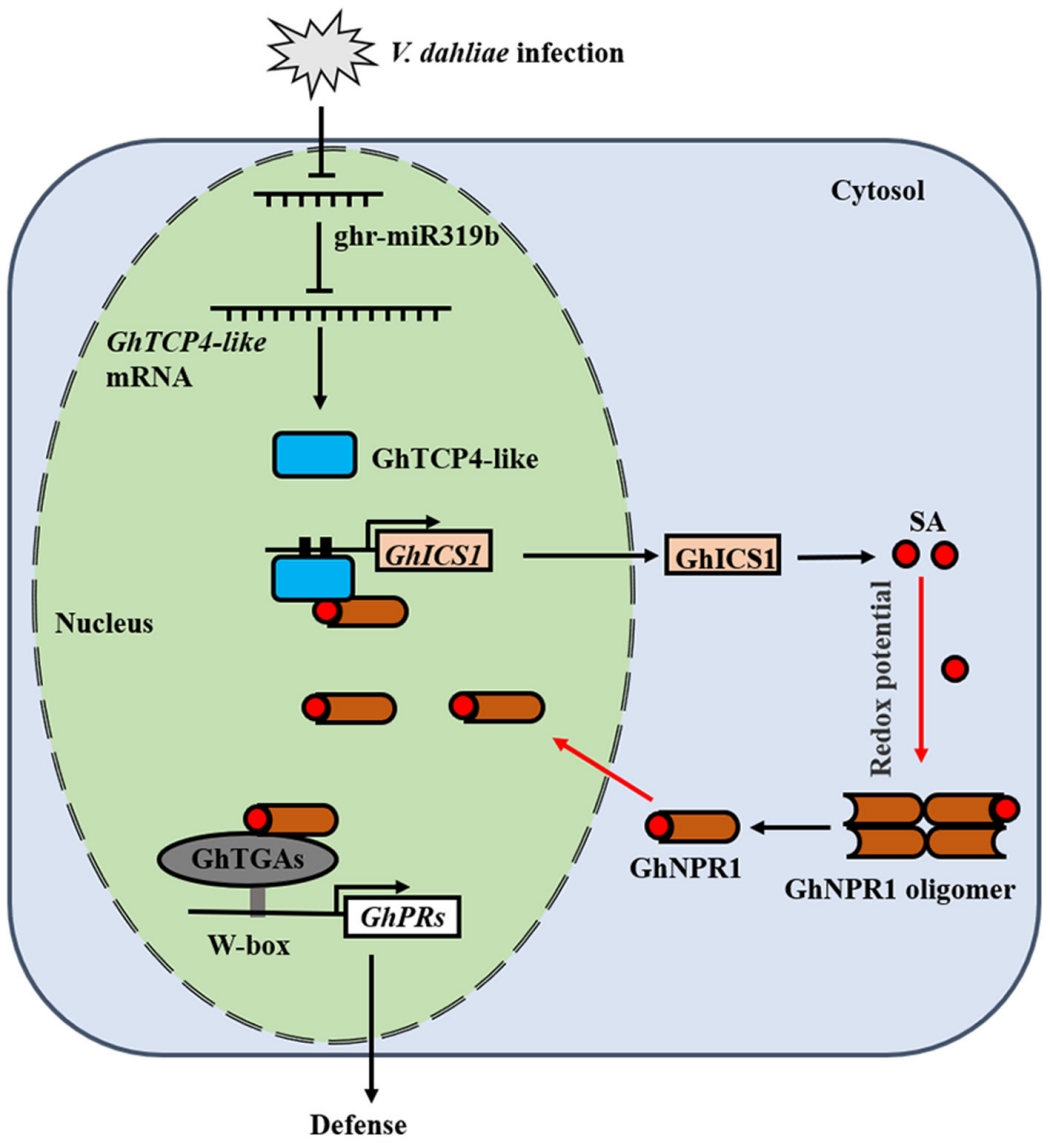
Working model diagram of ghr-miR319b-GhTCP4-like module in defense against *V. dahliae* infection. *V. dahliae* invasion releases the effectors or PAMPs to inhibit ghr-miR319, increasing GhTCP4-like expression, which transactivates *GhICS1* expression to promote SA biosynthesis. GhNPR1 interacting with GhTCP4-like enhances activation activity of GhTCP4-like to *GhICS1* transcription in feedback regulation loop of SA biosynthesis, facilitating in plant high resistance to *V. dahliae* infection. NPR1 interacting with TGAs promotes defense-related gene expression to increase plant resistance to pathogens. The black arrows represent regulation positively, the T-shaped arrows represent regulation negatively, the red arrows represent diffusion.

In the present study, we addressed *GhTCP4-like* targets of ghr-miR319b in response to *V. dahliae* infection. The Ghr-miR319b-*GhTCP4-like* module regulates plant resistance to pathogen challenge by regulating SA biosynthesis. GhTCP4-like interactions with GhNPR1 co-ordinately activate *GhICS1* transcription to promote SA accumulation, resulting in increased plant resistance to *V. dahliae* infection, possibly through GhNPR1 interaction with GhTGAs. Our results deepen the understanding of NPR1 interaction with TCPs in plant immunity through feedback regulation of SA biosynthesis (Figure 8).

## Data Availability Statement

The original contributions presented in the study are included in the article/Supplementary Material, further inquiries can be directed to the corresponding authors.

## Author Contributions

JW and QP conceived and designed the experiments. PJ, YT, and GH performed the experiments. PJ, YQ, and AC constructed the vectors and data analysis. PJ, NZ, and JW wrote the manuscript. All authors read and approved the final manuscript.

## Funding

This work was supported by the National Natural Science Foundation of China (31971905), and sponsored by State Key Laboratory of Cotton Biology Open Fund (CB2021B02).

## Conflict of Interest

YQ and AC were employed by Join Hope Seeds Co. Ltd. The remaining authors declare that the research was conducted in the absence of any commercial or financial relationships that could be construed as a potential conflict of interest.

## Publisher's Note

All claims expressed in this article are solely those of the authors and do not necessarily represent those of their affiliated organizations, or those of the publisher, the editors and the reviewers. Any product that may be evaluated in this article, or claim that may be made by its manufacturer, is not guaranteed or endorsed by the publisher.
