# Supplementary material for: Cotton miR319b-Targeted TCP4-Like Enhances Plant Defense Against Verticillium dahliae by Activating GhICS1 Transcription Expression
Source: Front Plant Sci. 2022 May 20;13:870882. doi: 10.3389/fpls.2022.870882 (PMC9164164; doi:10.3389/fpls.2022.870882)
Supplement: Supplementary file 1 [file Data_Sheet_1.ZIP › Supplementary Materials/Supplementary Figures.docx]

## **Supplementary Figures**

**
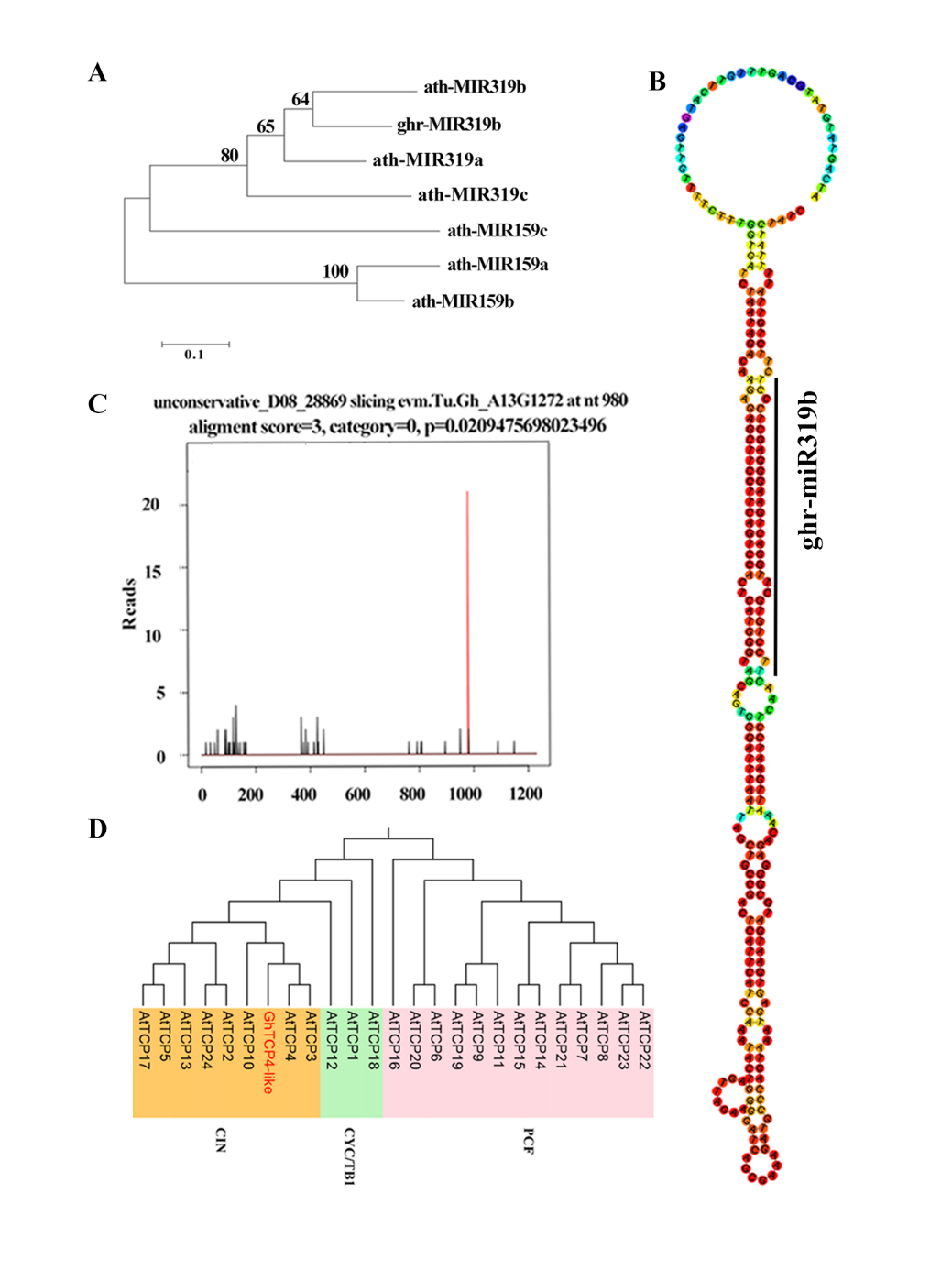
**

**Supplementary Figure S1.** Characterization and phylogenetic trees of ghr-miR319b and target gene *GhTCP4-like.* (A) phylogenetic tree analysis of *GhMIR319b* with *Ath-MIR319s* and *Ath-MIR159s*. (B) Predicting the secondary structure of GhMIR319b by RNA Fold program. The sequence marked by black solid line is the mature sequence of ghr-miR319b. (C) mRNA degradomes data showed that ghr-miR319b may guide Gh_A13G1272 transcript cleavage at 980-nt. (D) Phylogenetic tree analysis of GhTCP4-like with 24 other known TCPs of Arabidopsis. The neighbor-joining tree was constructed by MEGA7 program with 1000 bootstraps.


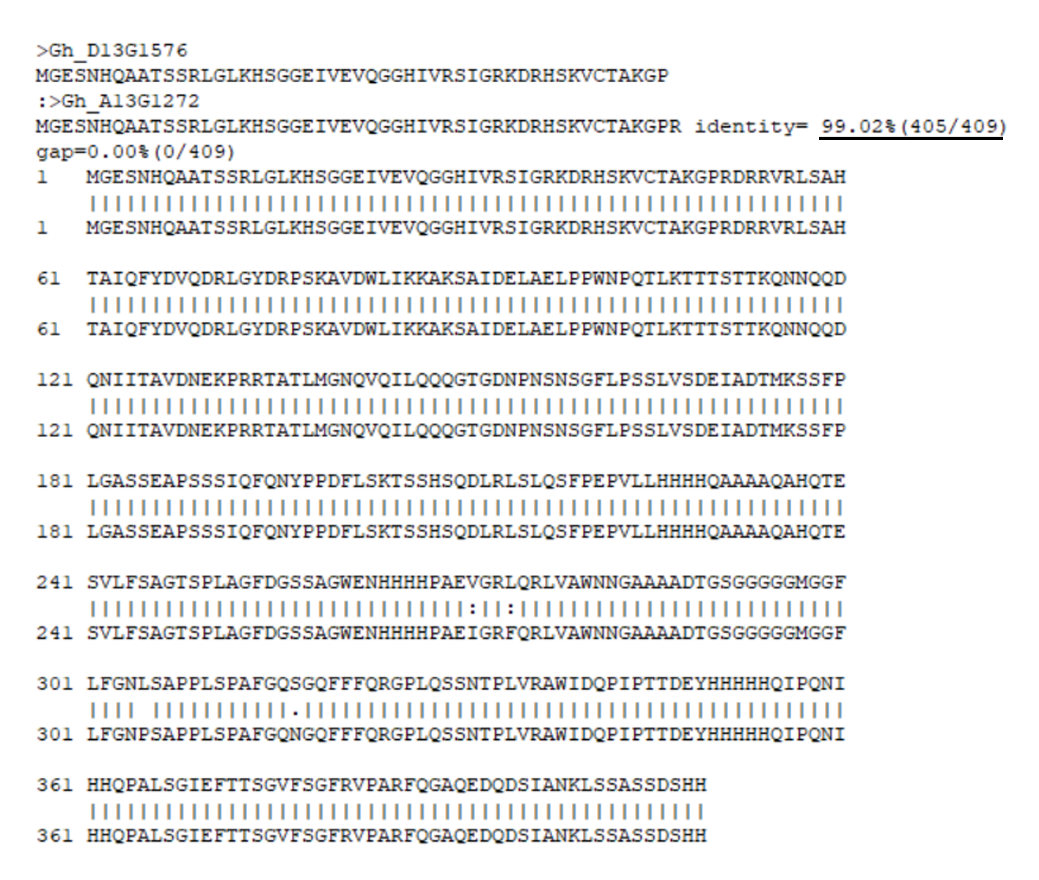


**Supplementary FIGURE S2.** Amino acid sequences alignment of GhTCP4-like-A (GhA_13G1272) and GhTCP4-like-D (GhD_13G1576) showing 99.02% identity.


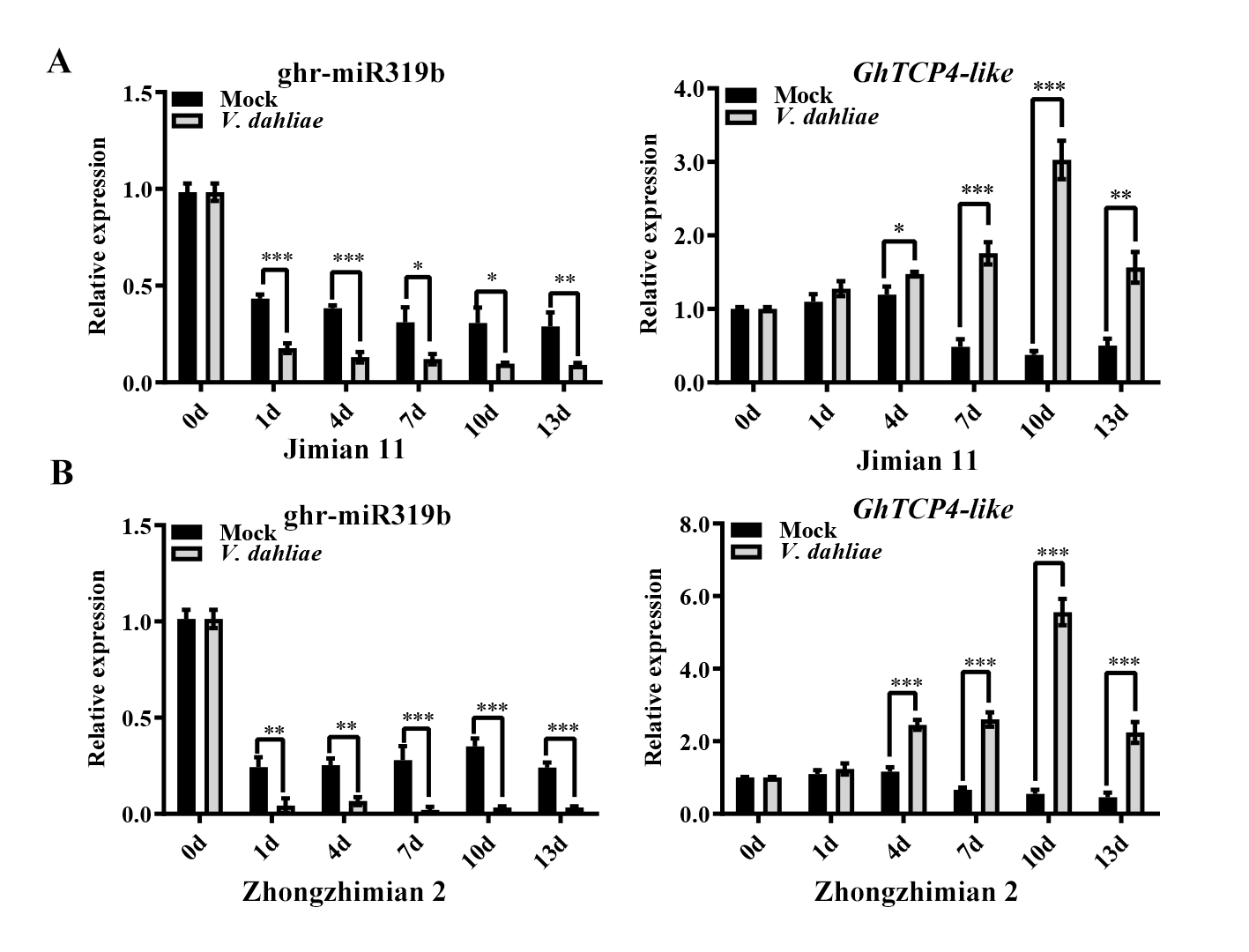


**Supplementary FIGURE S3.** Expression analysis of ghr-miR319b and *GhTCP4-like* in Zhongzhimian 2 and Jiammian 11 under *V. dahliae* infection. (A and B) Expression levels of ghr-miR319b and *GhTCP4-like* of Jimian 11 (A) and Zhongzhimian 2 (B) were detected by qPCR. Root samples were collected at 0, 1, 4, 7, 10, 13 days after *V. dahliae* infection. Error bar means SD of three independent biological replications. Student’s *t*-test was performed, **p* < 0.05, ***p* < 0.01, ****p* <0.001.


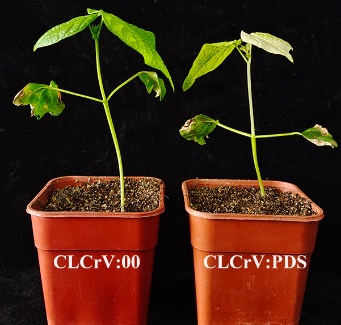


**Supplementary FIGURE S4.** Photobleaching phenotype of *PDS*-silenced plants. Photo was taken 14 days after agroinfiltration.


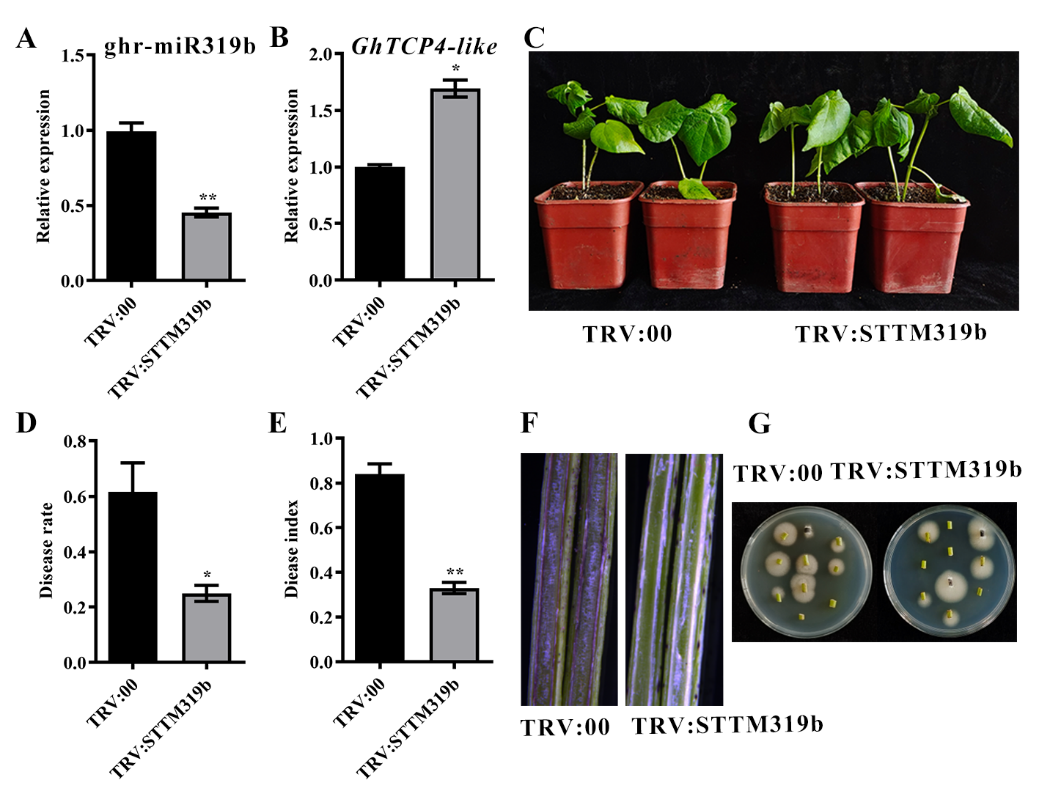


**Supplementary FIGURE S5.** Knockdown of ghr-miR319b increases cotton plant resistance to *V. dahliae* infection. (A) ghr-miR319b was silenced by TRV induced gene silencing. Expression of ghr-miR319b was analyzed by qPCR. Error bar means SD of three independent biological replications. Student’s *t*-test was performed, ***p* <0.01. (B) qPCR was performed to analysis *GhTCP4-like* expression. Error bar means SD of three independent biological replications. Student’s *t*-test was performed, **p* <0.05. (C) Disease symptoms of TRV:00 and TRV:STTM319b plants 14 days after *V. dahliae* infection. (D and E) Disease rate and disease index of TRV:00 and TRV:STTM319b plants were analyzed. Error bar means SD of three independent biological replications. Student’s *t*-test was performed, **p* <0.05. At least 30 seeding per treatment. (F) Colour intensity of longitudinal sections of stem of TRV:00 and TRV:STTM319b plants 21 dpi. Photos were taken by the body microscope. (G) Fungal recover assay. Photos were taken after 5 days of culture at 25°C.


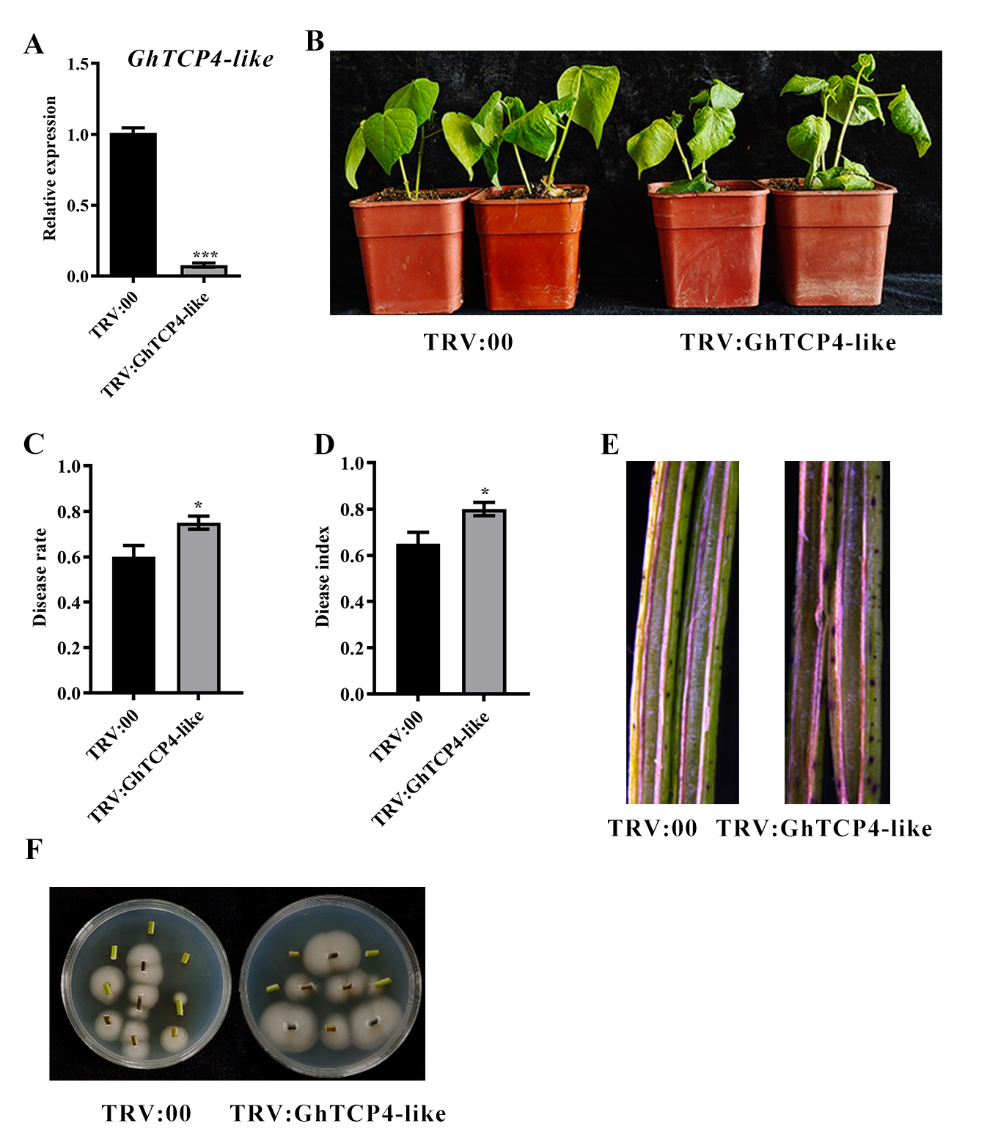


**Supplementary FIGURE S6.** *GhTCP4-like*-silenced plants were more susceptive to *V. dahliae* infection. (A) *GhTCP4-like* was silenced by TRV induced gene silencing. Expression of *GhTCP4-like* was analyzed by qPCR. Error bar means SD of three independent biological replications. Student’s *t*-test was performed, ****p* <0.001. (B) Disease symptoms of TRV:00 and TRV:GhTCP4-like plants 14 days after *V. dahliae* infection. (C and D) Disease rate and disease index of TRV:00 and TRV:GhTCP4-like plants was analyzed. Error bar means SD of three independent biological replications. Student’s *t*-test was performed, **p* <0.05. At least 30 seeding per treatment. (E) Colour intensity of longitudinal sections of stem of TRV:00 and TRV:GhTCP4-like plants 21 dpi. Photos were taken by the body microscope. (F) Fungal recover assay. Photos were taken after 5 days of culture at 25°C.
